# Comorbidities, Cardiovascular Therapies, and COVID-19 Mortality: A Nationwide, Italian Observational Study (ItaliCO)

**DOI:** 10.3389/fcvm.2020.585866

**Published:** 2020-10-09

**Authors:** Francesca Polverino, Debra A. Stern, Gaetano Ruocco, Elisabetta Balestro, Matteo Bassetti, Marcello Candelli, Bruno Cirillo, Marco Contoli, Angelo Corsico, Filippo D'Amico, Emilia D'Elia, Giuseppe Falco, Stefano Gasparini, Stefano Guerra, Sergio Harari, Monica Kraft, Luigi Mennella, Alberto Papi, Roberto Parrella, Paolo Pelosi, Venerino Poletti, Mario Polverino, Claudio Tana, Roberta Terribile, Jason C. Woods, Fabiano Di Marco, Fernando D. Martinez, The ItaliCO study group, F. Polverino

**Affiliations:** ^1^University of Arizona, Tucson, AZ, United States; ^2^Lovelace Respiratroy Research Institute, Albuquerque, NM, United States; ^3^Regina Montis Regalis Hospital, Mondovì, Italy; ^4^University Hospital of Padua, Padua, Italy; ^5^San Martino Hospital Istituto di Ricovero e Cura a Carattere Scientifico, Genoa, Italy; ^6^Agostino Gemelli University Polyclinic, Catholic University of the Sacred Heart, Rome, Italy; ^7^Sapienza University of Rome, Rome, Italy; ^8^University of Ferrara, Ferrara, Italy; ^9^University of Pavia, Pavia, Italy; ^10^Azienda Socio Sanitaria Territoriale Bergamo EST, Seriate, Italy; ^11^Ospedale Papa Giovanni XXIII, Bergamo, Italy; ^12^Azienda Unità Sanitaria Locale-Istituto di Ricovero e Cura a Carattere Scientifico di Reggio Emilia, Reggio Emilia, Italy; ^13^Marche Polytechnic University, Ancona, Italy; ^14^Department of Medical Sciences, San Giuseppe Hospital MultiMedica IRCCS, Milan, Italy; ^15^Department of Clinical Sciences and Community Health, University of Milan, Milan, Italy; ^16^San Luca Hospital, Vallo della Lucania, Italy; ^17^Department of Infectious Diseases, Colli Hospital, Naples, Italy; ^18^L. Pierantoni GB Morganis Hospital, Forlì, Italy; ^19^Mauro Scarlato Hospital, Azienda Sanitaria Locale Salerno, Scafati, Italy; ^20^ASL Lanciano Vasto Chieti, Chieti, Italy; ^21^University Hospital Major of Charity of Novara, Novara, Italy; ^22^Cincinnati Children's Hospital Medical Center, Cincinnati, OH, United States; ^23^University of Milan, Milan, Italy

**Keywords:** COVID-19, comorbidities, ACE inhibitors, mortality, cohort study

## Abstract

**Background:** Italy has one of the world's oldest populations, and suffered one the highest death tolls from Coronavirus disease 2019 (COVID-19) worldwide. Older people with cardiovascular diseases (CVDs), and in particular hypertension, are at higher risk of hospitalization and death for COVID-19. Whether hypertension medications may increase the risk for death in older COVID 19 inpatients at the highest risk for the disease is currently unknown.

**Methods:** Data from 5,625 COVID-19 inpatients were manually extracted from medical charts from 61 hospitals across Italy. From the initial 5,625 patients, 3,179 were included in the study as they were either discharged or deceased at the time of the data analysis. Primary outcome was inpatient death or recovery. Mixed effects logistic regression models were adjusted for sex, age, and number of comorbidities, with a random effect for site.

**Results:** A large proportion of participating inpatients were ≥65 years old (58%), male (68%), non-smokers (93%) with comorbidities (66%). Each additional comorbidity increased the risk of death by 35% [_adj_OR = 1.35 (1.2, 1.5) *p* < 0.001]. Use of ACE inhibitors, ARBs, beta-blockers or Ca-antagonists was not associated with significantly increased risk of death. There was a marginal negative association between ARB use and death, and a marginal positive association between diuretic use and death.

**Conclusions:** This Italian nationwide observational study of COVID-19 inpatients, the majority of which ≥65 years old, indicates that there is a linear direct relationship between the number of comorbidities and the risk of death. Among CVDs, hypertension and pre-existing cardiomyopathy were significantly associated with risk of death. The use of hypertension medications reported to be safe in younger cohorts, do not contribute significantly to increased COVID-19 related deaths in an older population that suffered one of the highest death tolls worldwide.

## Introduction

Italy, after Japan, tops the list of the world's oldest countries, with over 22% of its population aged 65 or older (1). Italy has been one of the hardest hit countries during the severe acute respiratory syndrome coronavirus 2 (SARS-CoV-2) pandemic. As of September 1 2020 over 35,500 persons had died due to Coronavirus disease 2019 (COVID-19), especially in the northern regions of the country, with 84.5% of deaths occurring in patients age 70 or older (Istituto Superiore di Sanitá, ISS, https://www.epicentro.iss.it/coronavirus/), and a crude case fatality rate in the region Lombardy of 18.3% (2). Previous study showed that comorbid conditions play a relevant role in increasing the risk of death in patients with COVID-19 (3–9). In particular, hypertension and underlying cardiovascular diseases (CVDs) have been strongly associated with death in COVID-19 inpatients (7, 10, 11), and case fatality rates tend to be high in older people and hypertensive individuals (12). Indeed, the prevalence of CVDs in COVID-19 patients across studies ranges from 8 to 42% (13). Hypertension, heart failure (HF) and ischemic heart disease are often treated with renin-angiotensin-aldosterone system (RAAS) blockers such as angiotensin-converting enzyme (ACE) inhibitors or angiotensin receptor blockers (ARBs). The use of ACE inhibitors/ARBs in patients with COVID-19 or at risk of infection with the virus is currently a subject of intense debate (14, 15), due to the evidence that SARS-CoV-2 uses the ACE2 receptor for entry into target cells (16). ACE2 and its related axis are an endogenous counter-regulatory system, with effects opposite to those of the ACE axis (17, 18). Nonetheless, ACE inhibitor/ARBs have also been associated with a reduction of mortality and re-hospitalization in patients with cardiovascular diseases due to their anti-thrombotic and anti-inflammatory effects, and protective effects against endothelial dysfunction (13). Whether the use of ACE inhibitors and ARB affects the mortality of COVID-19 patients has been debated. The only and largest survey published so far assessing the association between comorbidities, use of ACE inhibitors/ARBs, and COVID-19 death included 4,480 patients from Denmark (12). The authors found no evidence that either ACE inhibitors or ARB increased the risk for death among persons hospitalized for COVID-19. However, the COVID-19-related death burden in Denmark has been tremendously lower than Italy (628 vs. 35,595, respectively, as of September 1 2020) which poses questions on the heterogeneity of the Italian and Danish populations and the ways COVID-19 hit and was handled by the two countries. A second study from China also excluded subjects aged ≥75 years (19). To date, there is no study available of the relation between chronic use of ACE inhibitors and ARB, considered separately, and mortality in hospitalized COVID-19 patients that includes sufficient patients in the older age group, and that accounts for concomitant cardiovascular therapies or comorbid conditions. Moreover, due to higher fatality of COVID-19 infection in patients affected by CVDs, there is an unmet need to understand the link between cardiovascular therapies, CVD and COVID-19 severity and mortality.

In this study, we describe baseline characteristics and factors associated with death among 3,179 patients hospitalized for COVID-19, who were either discharged or died, and who were residents of 19 out of Italy's 21 regions, including the main islands. In assessing potential risks for death, we specifically assessed the comorbidities and the role of pre-hospitalization ACE inhibitors and ARBs and other commonly used CVD medications (such as beta-blockers, calcium channel blockers and diuretics). By age distribution, our sample is representative of older people, that are most severely affected by COVID-19 since the beginning of the pandemic (5, 6).

## Methods

### Patient Inclusion

**Figure 1:**
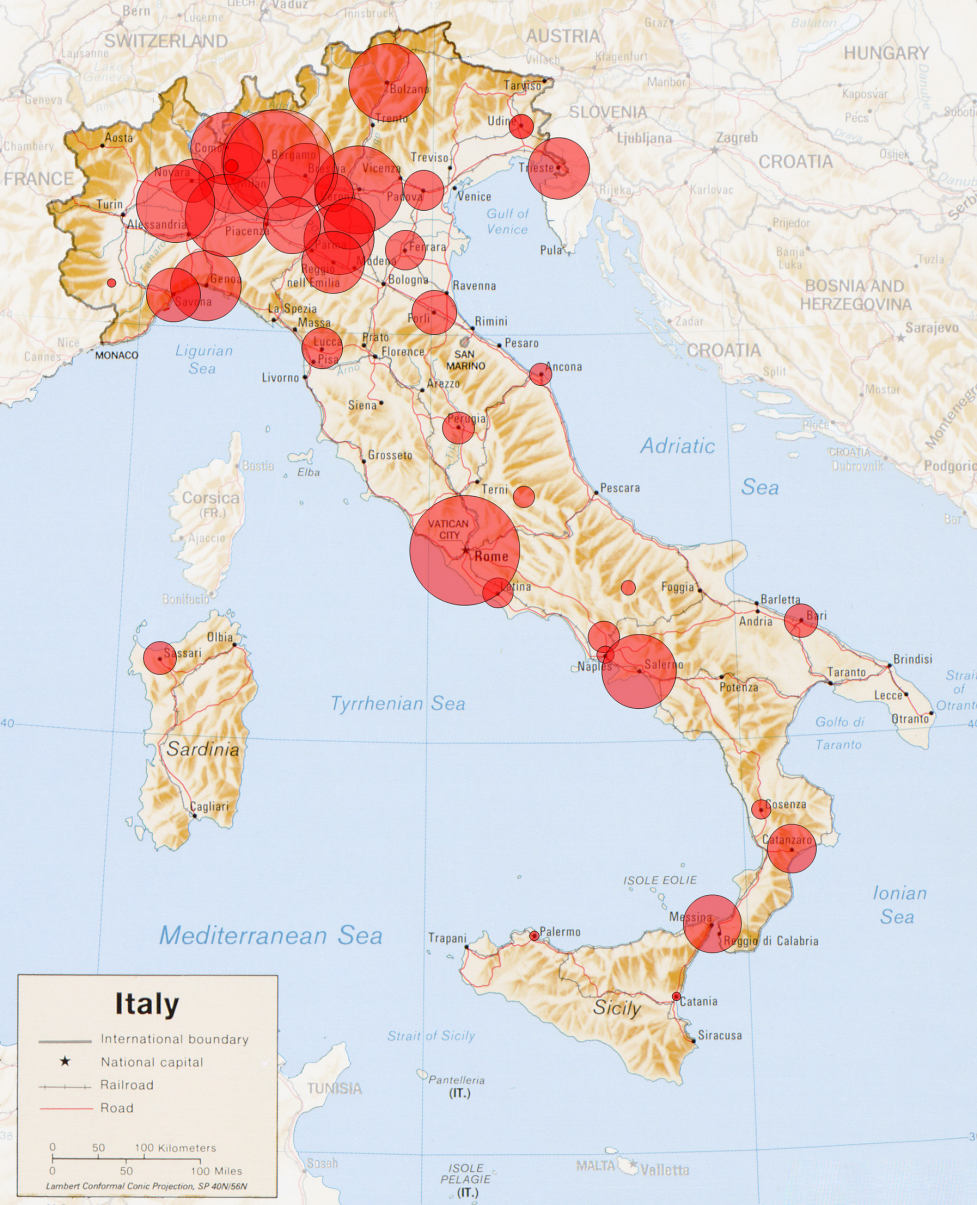
Italian Cartographic representation of the study subjects: Cartographic representation of the patients in this study cohort, with the area of each red circle proportional to the combined number of patients from each compact metropolitan area.

Data for 5,625 patients hospitalized for COVID-19 and with a positive nasopharyngeal swab for SARS-CoV-2 virus were manually extracted from medical charts from 61 hospitals across Italy. Patients were included in this analysis if they had been either discharged or had died at the time of ascertainment (*n* = 3,179, Figure 1, 56 sites). The status for each patient was reported at the time of data collection by the local investigators and represents an assessment of the patient's condition between March 25 and April 22, 2020. All the patients' information was obtained by manual review of the medical charts by the attending physician or nurse during their shifts. Each participating center was provided, upon enrollment, with a database to fill with patients' demographic, social, and clinical information and detailed instructions about the data collection. Smoking history was manually extracted from the chart for each patient. Information about smoking was not available for 316 patients. The collection and analysis of data in the registry have been deemed exempt from ethics review.

### Comorbidities

**Figure 2:**
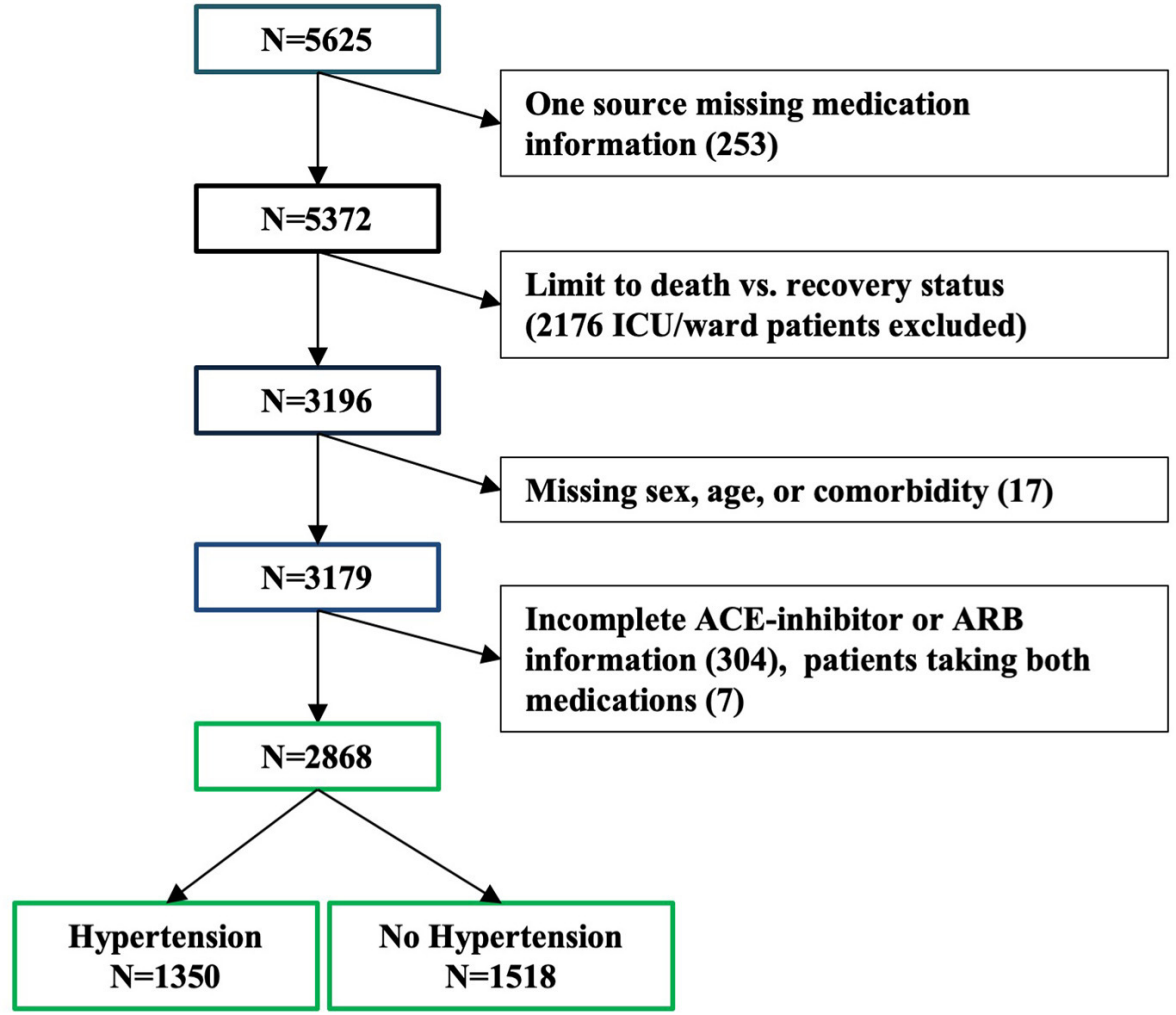
Flow chart of patient sample sizes.

Investigators manually extracted information about preexisting comorbidities known or suspected to be associated with COVID-19 mortality from the chart of each patient that was still hospitalized in their hospital or discharged within 30 days from the collection of the data. Information was available for atrial fibrillation, blood cancer, organ cancer, coronary artery disease, cardiomyopathy, chronic heart failure, chronic obstructive pulmonary disease (COPD), chronic renal failure, diabetes, hypertension, obesity, and stroke. We used a count of the reported number of comorbidities for each patient to assess their combined effect on mortality. Patients missing comorbidity information were excluded from these analyses (*n* = 17, Figure 2).

### Cardiovascular Medications

For this study, we specifically targeted extraction of detailed information from the patient's chart regarding use of ACE inhibitors and ARB at the time of admission. We also extracted information about other medications usually prescribed for hypertension (beta-blockers, diuretics, and Ca-antagonists).

### Statistics

**Figure 3:**
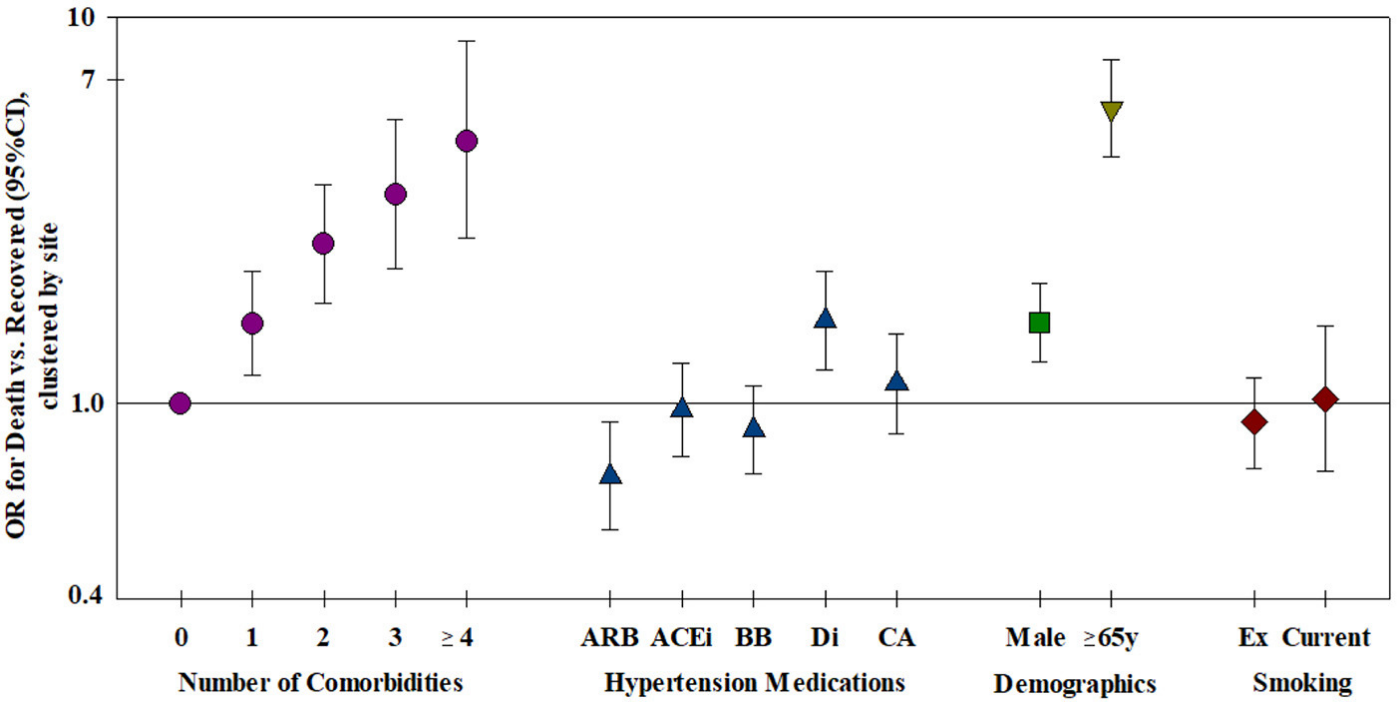
Risk factors for mortality—all risk factors were included in the model, clustered by site (*n* = 2,868). ARB, Angiotensin receptor blocker; ACEi, Angiotensin converting enzyme inhibitor; BB, Beta-blocker; Di, Diuretic; CA, Ca-antagonist.

A generalized linear mixed model, mixed-effects logistic regression, was used to assess the relations of sex, age, comorbidity count and hypertension medication use to death relative to recovery (STATA 16, StataCorp, College Station, TX, USA). The primary outcome was inpatient mortality. Since data were clustered by hospital site, site was included in the models as a random effect to account for potential within site correlation of patient characteristics. The number of patients contributed by each hospital site varied, ranging from 2 to 242 patients (Supplementary Figure 1). A dummy category for those patients missing smoking information was included in the model for Figure 3.

## Results

There were 3,179 patients with complete data for sex, age, status, and comorbidities (Table 1); 2,282 (71.8%) had been discharged from the hospital and 897 (28.2%) had died. The median age was 69.0 years, with an interquartile range of 57 to 78 years (Supplementary Figure 2).

**Table 1:** Characteristics of all patients, recovered patients and deceased patients.

**Characteristic**	**Group**	**All patients (*****n*** **=** **3,179)**		**Recovered (*****n*** **=** **2,282)**		**Deceased (*****n*** **=** **897)**	
		***N***	**%**	***N***	**%**	***N***	**%**
Sex	Female	1,008	31.7	764	33.5	244	27.2
	Male	2,171	68.3	1,518	66.5	653	72.8
Age	<20	10	0.3	10	0.4	0	0
	20 <30	35	1.1	34	1.5	1	0.1
	30 <40	102	3.2	101	4.4	1	0.1
	40 <50	260	8.2	250	11.0	10	1.1
	50 <60	547	17.2	497	21.8	50	5.6
	60 <70	707	22.2	572	25.1	135	15.1
	70 <80	828	26.1	525	23.0	303	33.8
	80+	690	21.7	293	12.8	397	44.3
Age	<65	1,320	41.5	1,205	52.8	115	12.8
	≥65	1,859	58.5	1,077	47.2	782	87.2
Each comorbidity^a^	Atrial fibrillation	256	8.1	142	6.2	114	12.7
	Blood cancer	28	0.9	19	0.8	9	1.0
	Coronary artery disease	359	11.3	186	8.2	173	19.3
	Cardiomyopathy	105	3.3	54	2.4	51	5.7
	Chronic heart failure	119	3.7	59	2.6	60	6.7
	COPD	188	5.9	98	4.3	90	10.0
	Chronic renal failure	157	4.9	72	3.2	85	9.5
	Diabetes	518	16.3	319	14.0	199	22.2
	Hypertension	1,500	47.2	960	42.1	540	60.2
	Obesity	218	6.9	163	7.1	55	6.1
	Organ cancer	135	4.3	82	3.6	53	5.9
	Stroke	107	3.4	52	2.3	55	6.1
Comorbidities^b^	0	1,096	34.5	939	41.2	157	17.5
Count	1	1,043	32.8	756	33.1	287	32.0
	2	644	20.3	388	17.0	256	28.5
	3	274	8.6	144	6.3	130	14.5
	≥4	122	3.8	55	2.4	67	7.5
Smoking^c^	Never	1,963	68.6	1,437	68.9	526	67.7
	Ex	692	24.2	496	23.8	196	25.2
	Current	208	7.3	153	7.3	55	7.1
Race^d^	Caucasian	2,983	97.5	2,133	96.6	850	99.8
	Not caucasian	77	2.5	75	3.4	2	0.2
Regional areas	Lombardia	1,397	43.9	901	39.5	496	55.3
	Northeastern	634	19.9	481	21.1	153	17.1
	Northwestern	380	12.0	272	11.9	108	12.0
	Central	400	12.6	340	14.9	60	6.7
	Southern	368	11.6	288	12.6	80	8.9

a *Percentage with each comorbidity was calculated as the number with the comorbidity divided by the total for that column*.

b *Number of comorbidities were summed for each patient and included: atrial fibrillation, blood cancer, organ cancer, coronary artery disease, cardiomyopathy, chronic heart failure, COPD, chronic renal failure, diabetes, hypertension, obesity and stroke*.

c *316 patients were missing smoking information*.

d *119 patients were missing race information*.

### Risk Factors for Death

The relation of age and death was non-linear; very few patients under the age of 50 died (Table 1). Males were more likely to die than females and patients aged ≥65 years were over six times more likely to die compared to younger patients (Table 2). Current smoking was unrelated to death in either univariate or multivariable analyses (Figure 3). Hypertension (47.2%) was the most frequent comorbidity, followed by diabetes (16.3%), coronary artery disease (11.3%), atrial fibrillation (8.1%), and obesity (6.9%) (Table 1). In a model including all comorbidities and adjusted for sex, age and site, the comorbidities cardiomyopathy, COPD, chronic renal failure, hypertension, obesity, organ cancer, and stroke were all independent risk factors for death (Table 3). Interestingly, while only 5.9% of COVID-19 inpatients had COPD, the latter was highly and significantly associated with increased risk of death (Table 3). In addition to evaluating the relation of the individual comorbidities to death, the count of comorbidities reported for each patient was strongly associated with risk of death (Table 2), with the odds for death increasing by 35% for each additional comorbidity (evaluated as an ordinal count; adjOR =1.35 [1.2, 1.5] *p* < 0.001), after adjusting for sex, age, and site.

**Table 2:** Proportion of patient groups who died vs. recovered at the time of data collection.

**Characteristic**	**Group**	**All patients (*****n*** **=** **3,179)**									
						**Univariate**			**Multivariable**		
		**Outcome**				**Death**			**Death**		
		**Total *N***	**Recovered *N***	**Deceased *N***	**Deceased %**	**OR^a^**	**95%CI**	***P***	**_adj_OR^c^**	**95%CI**	***P***
Sex	Female	1,008	764	244	24.2	ref			ref		
	Male	2,171	1,518	653	30.1	1.42	1.2, 1.7	0.001	1.56	1.3, 1.9	<0.001
Age	<65	1,320	1,205	115	8.7	ref			ref		
	≥65	1,859	1,077	782	42.1	8.25	6.4, 10.6	<0.001	6.35	4.9, 8.2	<0.001
Comorbidities^b^	0	1,096	939	157	14.3	ref			ref		
	1	1,043	756	287	27.5	2.59	2.0, 3.3	<0.001	1.71	1.3, 2.2	<0.001
	2	644	388	256	39.8	4.54	3.5, 6.0	<0.001	2.52	1.9, 3.4	<0.001
	3	274	144	130	47.5	6.69	4.7, 9.4	<0.001	3.29	2.3, 4.7	<0.001
	≥4	122	55	67	54.9	9.26	5.7, 14.9	<0.001	4.49	2.7, 7.4	<0.001

*Univariate and multivariable estimates for the relation of sex, age, and number of comorbidities to patient mortality, with clustering for site*.

a *Odds ratio for death estimated with clustering for site (56 sites)*.

b *Number of comorbidities were summed for each patient and included: atrial fibrillation, blood cancer, organ cancer, coronary artery disease, cardiomyopathy, chronic heart failure, COPD, chronic renal failure, diabetes, hypertension, obesity and stroke*.

c *Multivariable model included sex, age divided into those <65 and those ≥65 years old, and the number of comorbidities as a categorical covariate with clustering for site as a random effect*.

**Table 3:** Multivariable model for the risk of death associated with each comorbidity after adjustment for sex and age, clustering for site as a random effect.

**Comorbidity**	**All patients (*****n*** **=** **3,179)**		
	**Risk of death**		
	**_**adj**_OR**	**95%CI**	***P***
Atrial fibrillation	0.97	0.69, 1.36	0.874
Blood Cancer	0.93	0.29, 2.97	0.901
Coronary artery disease	1.11	0.83, 1.49	0.471
Cardiomyopathy	**1.85**	**1.11, 3.11**	**0.019**
Chronic heart failure	0.74	0.44, 1.23	0.240
COPD	**1.93**	**1.31, 2.85**	**0.001**
Chronic renal failure	**1.71**	**1.09, 2.67**	**0.019**
Diabetes	1.21	0.93, 1.58	0.149
Hypertension	**1.24**	**1.00, 1.53**	**0.049**
Obesity	**2.03**	**1.30, 3.17**	**0.002**
Organ cancer	**1.67**	**1.04, 2.68**	**0.032**
Stroke	**2.00**	**1.22, 3.27**	**0.006**
Male	**1.85**	**1.47, 2.33**	** <0.001**
Age, years	**1.10**	**1.09, 1.11**	** <0.001**

*P <0.05 are indicated in bold*.

### Analysis of Variability Across Geographic Regions and Hospitals

There were differences in the number of comorbidities across the hospitals and regional areas that provided data (Supplementary Table 1). Patients from the Central Italy were reported to have the fewest number of comorbidities and those from the Northeastern region to have the most. Therefore, in order to assess whether the geographic/hospital variation in diagnostic labeling of comorbidities could have influenced the meaning of the associations made between comorbidities and mortality, in addition to the random effect adjustment for hospital site, we added regional area to the multivariable model shown in Table 2. There was no appreciable change in the relation of any risk factor to death, including comorbidity count, after this additional adjustment (data not shown).

### Risk of Death by Hypertension Medication Use

Of the 3,179 patients, 2,868 had complete information for hypertension medication use. Patients with no comorbidities were less likely to use ARBs and ACE inhibitors but there was no trend for increased use of ACE inhibitors or ARBs among patients with one or more comorbidities (Supplementary Table 2). The use of diuretics, beta-blockers and Ca-antagonists increased significantly with the number of comorbidities reported for each patient. Comorbidities were strongly associated with age but not with sex (Supplementary Table 2).

Most of the 951 patients taking either ACE inhibitors or ARB at admission had hypertension, 87.9 and 90.3%, respectively, and beta-blocker use was reported for 29.9%, diuretic use for 24.3% and Ca-antagonist use for 22.8%. After adjustment for age, sex, number of comorbidities, smoking and site, we found no increased risk of death associated with the use of ACE inhibitors, ARBs, beta-blockers or Ca-antagonists (Figure 3 and Table 4). There was a marginal negative association between ARB use and a marginal positive association between diuretic use and death (Figure 3, *p* = 0.025 and *p* = 0.020, respectively).

**Table 4:** List of risk factors included in the model, clustered for site as a random effect.

**Characteristic**	**Group**	**Patients with complete hypertension medication information (*****n*** **=** **2,868)**						
						**Death**		
		**Total *N***	**Recovered *N***	**Deceased *N***	**Deceased %**	**_**adj**_OR**	**95%CI**	***P***
Comorbidities^b^	0	982	845	137	14.0	ref		
	1	927	690	237	25.6	1.64	1.19, 2.24	0.020
	2	595	365	230	38.7	2.64	1.85, 3.78	<0.001
	3	250	129	121	48.4	3.56	2.29, 5.55	<0.001
	≥4	114	50	64	56.1	4.88	2.73, 8.72	<0.001
ARB	No	2,446	1,767	679	27.8	ref		
	Yes	422	312	110	26.1	0.65	0.47, 0.89	0.025
ACE inhibitor	No	2,339	1,742	597	25.5	ref		
	Yes	529	337	192	36.3	0.97	0.73, 1.29	0.929
Beta-blocker	No	2,247	1,702	545	24.3	ref		
	Yes	621	377	244	39.3	0.85	0.65, 1.12	0.244
Diuretic	No	2,456	1,870	586	23.9	ref		
	Yes	412	209	203	49.3	1.66	1.23, 2.25	0.020
Ca-antagonist	No	2,488	1,842	646	26.0	ref		
	Yes	380	237	143	37.6	1.13	0.84, 1.53	0.773
Sex	Female	904	684	220	24.3	ref		
	Male	1,964	1,395	569	29.0	1.65	1.29, 2.09	<0.001
Age	<65years	1,207	1,100	107	8.9	ref		
	≥65 years	1,661	979	682	41.1	5.92	4.47, 7.83	<0.001
Smoking^c^	No	1,771	1,293	478	27.0	ref		
	Ex	642	470	172	26.8	0.89	0.68, 1.17	0.412
	Current	187	135	52	27.8	1.04	0.66, 1.62	0.879
	Unknown	268	181	87	32.5	1.65	1.08, 2.52	0.020

*The risk factors for mortality are also listed in Figure 3*.

^a^*Multivariable model included sex, age, number of comorbidities as a categorical covariate, smoking and each hypertension medication, with clustering for site as a random effect (55 sites); a dummy category for patients with missing smoking history was included in the model*.

b *Number of comorbidities were summed for each patient and included: atrial fibrillation, blood cancer, organ cancer, coronary artery disease, cardiomyopathy, chronic heart failure, COPD, chronic renal failure, diabetes, hypertension, obesity and stroke*.

c *Patients who were missing information about smoking were included as a separate smoking category in the model*.

## Discussion

This is the first and largest Italian countrywide study to date of COVID-19 inpatients, the majority of which was aged over 65, who either died or were discharged from hospital in 19 out of the 21 Italian regions, including the major islands (Figure 1). We found, in line with previous publications (3–9), that pre-existing comorbidities are major risk factors for death in COVID-19 patients. We report that the number of comorbidities is linearly and strongly associated with the risk of COVID 19-related death. However, after adjusting for comorbidities, age, and sex, we report that the lack of an association between risk of inpatient death due to COVID-19 and use of CVD medications reported in younger patients (3–9), extends to the older population at highest risk for COVID-19. Diuretics were associated with a marginal increased risk of death, and ARBs with a marginal decreased risk of death in this sample. Each comorbidity increased mortality risk independently from age and gender. Among CVD, cardiomyopathies and hypertension were related to a poor outcome. These findings are in line with Inciardi et al. (20) who showed that, in the Northern Italian population, COVID-19 patients with pre-existing CVD had an increased rate of death compared to patients without CVD. The underlying systemic inflammation in patients with CVD (21) might contribute to the increase immune responses and inflammatory cascade known to lead to a worse prognosis in COVID-19 patients (22).

Prior therapy with ACE inhibitors/ARBs was not related to worse prognosis in this cohort. The use of ACE inhibitors and ARB in patients with COVID-19 has been called into question by some (14), due to the evidence that SARS-CoV-2 uses the ACE2 receptor for entry into target cells (16). On the other hand, it has been recently shown that treatment with ACE inhibitors and ARBs does not increase ACE2 plasma levels in patients with heart failure (23). A recent study by Fosbol et al. (12) showed no association between risk of mortality in the Danish population and ACE inhibitor/ARB use. However, the COVID-19-related death burden in Denmark has been ~57-fold lower than Italy (https://coronavirus.jhu.edu/data/mortality), which calls for further studies investigating the characteristics of the Italian COVID-19 population. Additionally, Fosbol et al. did not compare the use of ACE inhibitors to the use of ARB, and the data were computed from national electronic health record review. Here we report that a similar conclusion is applicable to the Italian population. As the north of Italy, and especially the region Lombardia, suffered from one of the highest COVID-19 mortality rates worldwide (2), our data (carefully collected by manual review of medical charts from Italian 56 hospital distributed throughout the peninsula) are particularly important in order to understand the characteristics of the Italian COVID-19 patients with regional specificity. Also, in our study, a higher number of inpatients taking either ACEi or ARB than the Danish cohort was enrolled, thus allowing a stronger statistical power to rule out the individual effects of ACE inhibitors and ARB treatments. In another study, Reynolds et al. reported that previous treatment with ACE inhibitors or ARBs was not associated with a higher risk of testing positive for COVID-19 (10). Similarly, Mancia et al. recently showed that the use of ARBs and ACE inhibitors was more frequent among patients from one Italian region (Lombardy) who were infected with SARS-CoV-2 than among a large population of controls who were matched for age, sex, and place of residence (9). However, in the same study, neither ACE inhibitors nor ARBs showed an independent association with COVID-19 in patients with mild-to-moderate disease or in those with severe disease. Neither of these two latter studies had mortality as primary outcome, but rather the likelihood of the subjects of testing positive for COVID-19, or experiencing severe manifestations of COVID-19.

In a study of 5,700 patients hospitalized with COVID-19 in the New York City area, the mortality rates for patients with hypertension not taking an ACE inhibitors or ARBs at admission, taking an ACE inhibitor, or taking an ARB were comparable (8). Moreover, among 1,128 hospitalized COVID-19 patients with hypertension from Hubei, China, the inpatient use of ACE inhibitors/ARB was reported to be associated with lower risk of all-cause mortality compared with ACE inhibitors/ARB non-users (19). However, in the first study (8) the results were unadjusted for known confounders, including age, sex, race, ethnicity, and comorbidities. In the second study (19), the sample-size included only 188 patients who received ACE inhibitors/ARB, and thus it did not have the power to test the effects of ACE inhibitors and ARBs separately.

This is the first study assessing the safety of CVD medications, and in particular ACE inhibitors and ARB studied separately, in a nation-wide representative sample of COVID-19 inpatients, mostly aged >65. We provide strong evidence suggesting that, regardless of a person's risks for COVID19, the five drugs most frequently used for the treatment of CVDs in outpatient settings are not associated with increased inpatient mortality due to COVID-19.

Among CVD therapies, diuretics were associated with a marginally significant increased risk of mortality in our final models. However, the proportion of patients who were using diuretics at the time of admission increased markedly with the number of comorbidities in each patient. Although we adjusted for number of comorbidities, it is likely that residual confounding may be present. Similarly, use of ARBs was associated with a small decrease in death rates after adjustment for confounders, but the effect was marginally significant and may have been influenced by unaccounted confounding.

Our study has several strengths: (1) it is the first Italian country-wide study describing the characteristics of a highly heterogeneous cohort of COVID-19 inpatients both in terms of severity of the clinical manifestations, and also in terms of geographical distribution. We believe that the analyses performed in this study are clinically informative given that the north of Italy suffered from one of the highest mortalities for COVID-19 worldwide; and (2) the availability of a sample representative of the older people (>65 years of age) at highest risk for morbidity and mortality due to COVID-19. Limitations include: (1) the observational nature of the study, and the fact that data on comorbidities were collected by manual review of the medical charts with no objective assessment; this methodological limitation did not allow the disentanglement of the independent associations of antihypertensive medications with mortality (i.e., adequate control for disease related-, hypertension-, and other medication use- related variables); (2) lack of other outcome measures apart from death or hospital discharge, and of information about the cause of death of the patients (such as severe respiratory distress, acute kidney injury, myocardial infarction, pulmonary, and systemic thromboemobilsm); (3) the absence of information about specific treatments received during hospitalization and in-hospital ACE inhibitors/ARBs continuation or discontinuation; (4) the lack of a severity score for COVID-19 patients during hospitalization; and (5) the potential bias introduced by excluding patients that were still hospitalized at the time of data collection. However, we felt that including the latter could have introduced an even bigger bias, because these individuals could have had associations between the variables analyzed and the outcome that could be different from patients that were included because they had an outcome.

In summary, the results of our nationwide Italian study of a population of COVID19 inpatients, that suffered from one of the highest mortality rates worldwide, confirms that the number of comorbidities appears to be independently associated with increased COVID-19-related death. Among cardiovascular comorbidities both hypertension and pre-existing cardiomyopathy were associated with COVID-19 risk of death. Nonetheless, we provide reassuring evidence that use of commonly-used CVD medications is not associated with increased risk of death due to COVID-19, regardless of the person's risk for the disease due to age, sex, or comorbid conditions. Our findings show that there is no need to interrupt treatments for CVD in COVID-19 patients; in particular, the treatment with ACE inhibitors/ARBs should be continued in order to reduce potential cardiovascular derangement in COVID-19 patients. Further studies are needed in order to shed light onto the relationship between CVD therapies, underlying CVD, and prognosis in COVID-19 patients.

## Data Availability Statement

All datasets generated for this study are included in the article/Supplementary Material.

## Ethics Statement

The studies involving human participants were reviewed and approved by University of Arizona IRB waiver #2003521629. The ethics committee approvals/waivers were also obtained from each of the participating hospitals. The ethics committee waived the requirement of written informed consent for participation.

## Author Contributions

FP, GR, EB, MB, MCa, BC, MCo, AC, FD'A, ED'E, GF, SGa, SGu, SH, MK, LM, AP, RP, PP, VP, MP, CT, and RT collected the data. FP, DAS, SGu, JCW, and FDiM analyzed the data and wrote the manuscript. All authors contributed to the article and approved the submitted version.

## Conflict of Interest

MB has served on advisory boards and has received travel fundings, honoraria for speaking from Angelini, Astra Zeneca, bayer, Cubist, Pfizer, Menarini. MCo has received personal fees from Chiesi, AstraZeneca, Boehringer-Ingelheim, Alk-Abello, GSK, Novartis, Zambon, and scientific grants from Chiesi and University of Ferrara, Italy. FDiM has received personal fees from Chiesi, AstraZeneca, Boehringer-Ingelheim, GSK, Novartis, Zambon, Guidotti/Malesci, Menarini, Mundipharma, TEVA, Almiral, Levante Pharma, Sanophi, and scientific grants from AstraZeneca, Boehringer-Ingelheim, GSK, Novartis. AP has received board membership and consultancy fees, payment for lectures, grants for research, travel expenses reimbursements from GSK, AZ, Boehringer Ingelheim, Chiesi Farmaceutici, TEVA < Mundipharma, Zambon, Novartis, Menarini, Sanofi,Roche, Edmondpharma, Fondazione Maugeri, Fondazione Chiesi. JW has received investigator initiated research funding from Vertex pharmaceuticals. The remaining authors declare that the research was conducted in the absence of any commercial or financial relationships that could be construed as a potential conflict of interest.
