# Erratum: Comorbidities, Cardiovascular Therapies, and COVID-19 Mortality: A Nationwide, Italian Observational Study (ItaliCO)

**DOI:** 10.3389/fcvm.2020.631602

**Published:** 2020-12-09

**Authors:** Frontiers Production Office

**Affiliations:** Frontiers Media SA, Lausanne, Switzerland

**Keywords:** COVID-19, comorbidities, ACE inhibitors, mortality, cohort study

Due to a production error, **The consortium was not properly credited with authorship**. The publisher apologizes for this mistake. The original article has been updated.

