# Supplementary material for: Comorbidities, Cardiovascular Therapies, and COVID-19 Mortality: A Nationwide, Italian Observational Study (ItaliCO)
Source: Front Cardiovasc Med. 2020 Oct 9;7:585866. doi: 10.3389/fcvm.2020.585866 (PMC7583635; doi:10.3389/fcvm.2020.585866)

**Supp Table 1 Comorbidies by regional area in Italy**

| **Regional Area** |  | **Number of Comorbidities^1^** | | | | |
| --- | --- | --- | --- | --- | --- | --- |
|  | **n** | **0** | **1** | **2** | **3** | **≥4** |
| **Lombardia** | 1397 | 32.4 | 33.2 | 22.7 | 7.6 | 4.1 |
| **Northeastern** | 634 | 29.7 | 32.7 | 21.9 | 11.8 | 3.9 |
| **Northwestern** | 380 | 33.7 | 29.2 | 23.2 | 8.7 | 5.3 |
| **Central** | 400 | 51.0 | 30.8 | 9.5 | 7.5 | 1.3 |
| **Southern** | 368 | 33.4 | 37.5 | 16.9 | 8.2 | 4.1 |

^1^ Number of comorbidities were summed for each patient and included: atrial fibrillation, blood cancer, organ cancer, coronary artery disease, cardiomyopathy, chronic heart failure, COPD, chronic renal failure, diabetes, hypertension, obesity and stroke

**Supp Table 2: Characteristics of comorbidity count groups^1^**

|  |  |  |  | |  |  | **Hypertension Mediations** | | | | |
| --- | --- | --- | --- | --- | --- | --- | --- | --- | --- | --- | --- |
| **Number** |  |  | **Age** | | **Age ≥65** | **Male** | **ARB** | **ACEi** | **Diuretic** | **Beta-blocker** | **Ca-antagonist** |
| **of** |  | **n** | **mean** | **SD** | **%** | **%** | **%** | **%** | **%** | **%** | **%** |
| **Comorbidities**^1^ | **0** | 982 | 58.1 | 14.9 | 31.9 | 66.6 | 0.7 | 1.2 | 0.6 | 1.4 | 0.5 |
|  | **1** | 927 | 67.7 | 13.0 | 61.6 | 69.8 | 20.6 | 23.7 | 11.8 | 22.8 | 16.6 |
|  | **2** | 595 | 73.2 | 11.6 | 76.8 | 68.4 | 24.7 | 29.4 | 25.4 | 37.8 | 19.8 |
|  | **3** | 250 | 75.8 | 10.4 | 87.2 | 71.2 | 22.4 | 34.4 | 36.0 | 44.4 | 26.8 |
|  | **≥4** | 114 | 77.5 | 10.1 | 89.5 | 68.4 | 18.4 | 31.6 | 49.1 | 52.6 | 31.6 |
| **P** |  | 2868 | <0.001 |  | <0.001 | 0.523 | <0.001 | <0.001 | <0.001 | <0.001 | <0.001 |

^1^ Number of comorbidities were summed for each patient and included: atrial fibrillation, blood cancer, organ cancer, coronary artery disease, cardiomyopathy, chronic heart failure, COPD, chronic renal failure, diabetes, hypertension, obesity and stroke

**Supp Figure 1. The number of patients contributed by each clinical site, n=3179, 56 sites**


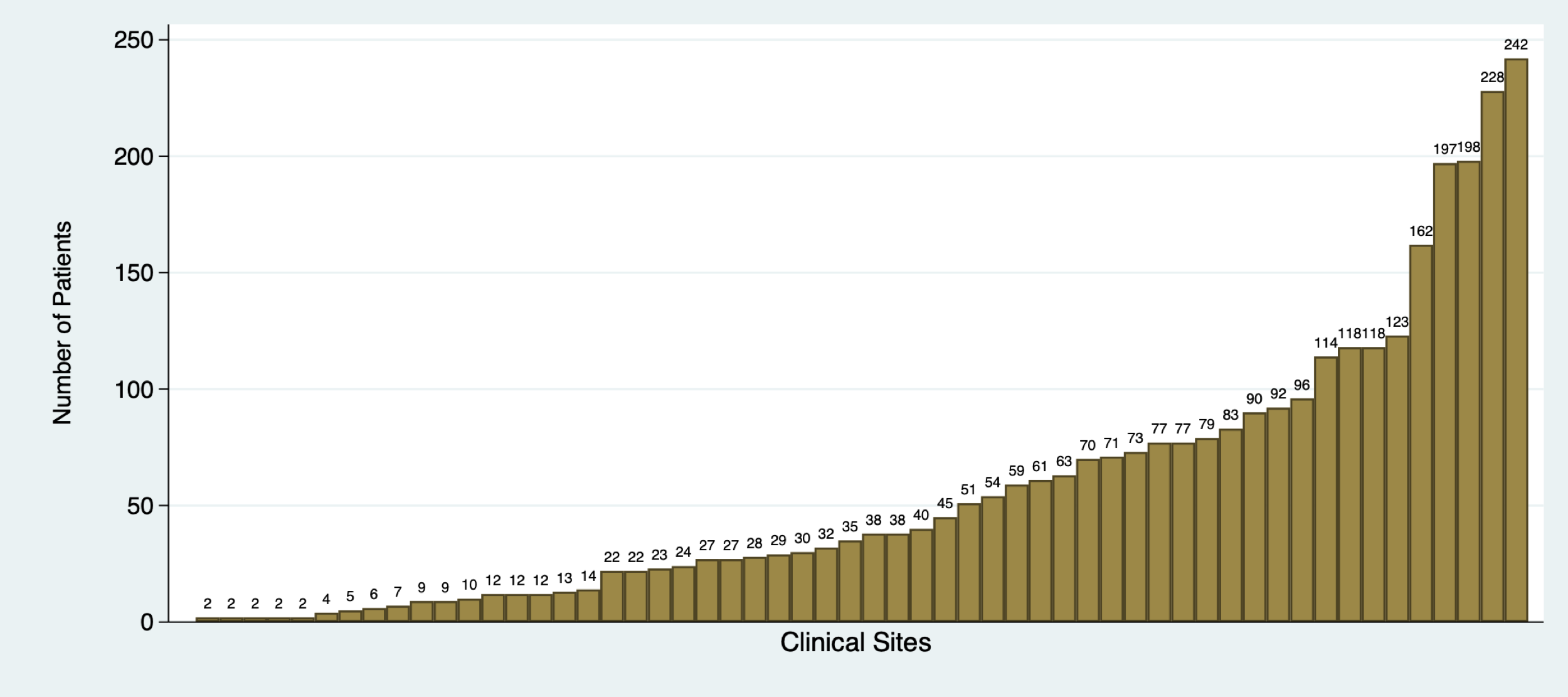


**Supp Figure 2. Histogram of patient age (n=3179)**


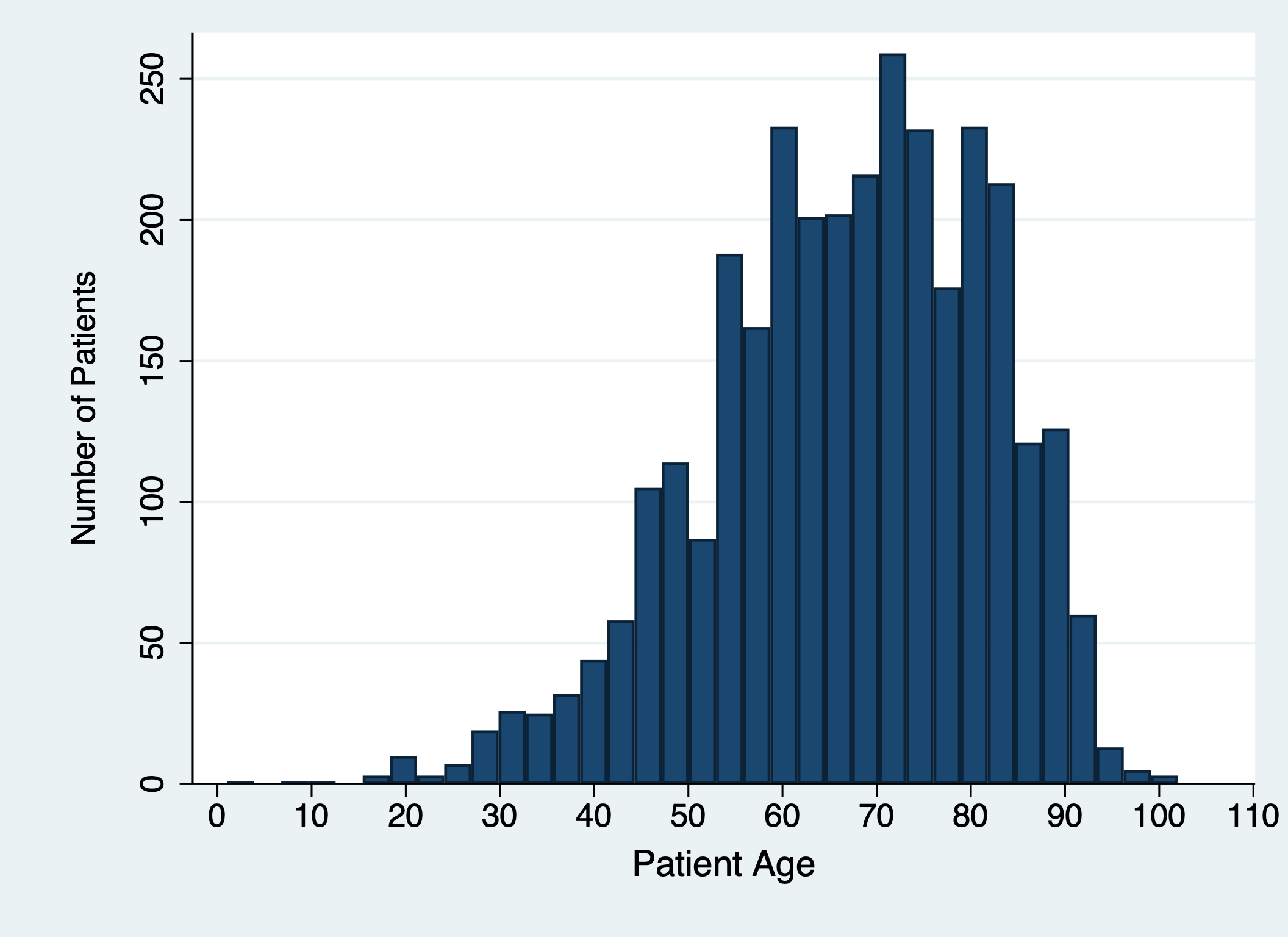

Supplement: Supplementary file 1 [file Table_1.docx]
