# Supplementary material for: Comorbidities, Cardiovascular Therapies, and COVID-19 Mortality: A Nationwide, Italian Observational Study (ItaliCO)
Source: Front Cardiovasc Med. 2020 Oct 9;7:585866. doi: 10.3389/fcvm.2020.585866 (PMC7583635; doi:10.3389/fcvm.2020.585866)
Supplement: Supplementary file 2 [file Data_Sheet_1.DOCX]

**ItaliCO: Italian national study on risk factors associated with COVID-19**

**List of Contributors:**

F. Polverino MD PhD^1^, D. Stern MS^1^, M. Polverino MD^2^, F. D’Amico MD^3^, E. D’Elia, MD PhD^4^, A. Agarossi MD^5^, S. Agati MD^6^, E. Agosteo MD^7^, F. Ando’ MD^8^, M. Andreoni MD^9^, IF. Angelillo DDS MPH^10^, G. Arcoleo MD^11^, C. Arena MD^12^, P. Baiamonte MD^11^, E. Balestro MD^13^, L. Ball MD, PhD^14^, P. Banfi MD^15^, G. Bartoletti MD^16^, R. Bartolotta RN^17^, M. Bassetti MD PhD^14^, D. Battaglini MD^14^, M. Bellan MD PhD^18^, I. Benzoni MD PhD^19^, R. Bertolini MD^20^, M. Bevilacqua MD^21^, M. Bezzi^22^ MD, A. Bianco MD^23^, A. Bisbano MD^24^,F. Bobbio MD^18^, G. Bocchialini MD^22^, F. Bonetti MD^20^, F. Boni MD^25^, M. Bonifazi MD^26^, G. Borgonovo MD^25^, S. Borre’ MD^27^, M. Bosio MD^28^, G. Brachini MD^29^, I. Brunetti MD^14^, L. Calagna^3^, F. Calò^10^, M. Candelli MD PhD^30^, A. Capuozzo MD^2^, T. Carr MD^1^, A. Castellani MD^22^, F. Catalano MD PhD^11^, G. Catania MD^31^, E. Catena MD^5^, M. Cattaneo^32^, A. Cattelan MD^13^, V. Ceruti MD^21^, F. Chiumiento MD^32^, G. Cicchitto MD^2^, B. Cirillo MD^29^, M. Confalonieri MD^34^, P. Confalonieri MD^34^, M. Contoli MD PhD^35^, N. Coppola MD PhD^10^, A. Corsico MD^28^, R. Cosentina MD^3^, R. Costantino MD^36^, C. Crimi MD PhD^37^, A. Currà MD^38^, M. D’Abbraccio MD^39^, A. Dalbeni MD^21^, F. Daleffe MD^22^, R. Davide MD^40^, M. Del Donno MD^41^, F. Di Marco, MD PhD^42^, F. Di Pastena MD^43^, F. Di Perna MD^44^, Z. Di Rosa MD^45^, A. Di Sabatino MD^28^, O. Elesbani MD^40^, D. Elia MD^32^, V. Esposito MD PhD^46^, L. Fabiani MD^47^, G. Falco MD^25^, G. Falo MD^35^, C. Fanelli MD^48^, A. Fantin MD^49^, F. Ferrigno MD^50^, G. Fiorentino MD^46^, F. Franceschi MD PhD^30^, M. Fronza MD^51^, G. Gardini Gardenghi MD^40^, S. Gasparini MD^26^, D.R. Giacobbe MD^14^, C. Giannotti MD^5^, G. Giannotti MD^19^, A. Gidari MD^52^, F. Giovanardi MD PhD^25^, P. Gnerre MD^31^, F. Gonnelli MD^26^, M. Graziano MD^53^, S. Greco MD^47^, A. Grosso PhD^28^, S. Guarino MD^32^, S. Guerra MD PhD^1^, S. Harari MD^32^, A. Iannarelli MD^54^, P. Imitazione MD^39^, F. Inglese MD^55^, V. Iodice MD^46^, A. Izzo MD^43^, C. La Greca MD^16^, M. Kraft MD^1^, A. Lax MD^15^, F. Legittimo MD^20^, A. Leo MD^56^, S. Leone MD^53^, V. Lepidini MD^9^, M. Leto RN^36^, F. Licata MD^23^, F. Locati MD^3^, L. Lorini MD^4^, B. Lucchetti MD^20^, I. Maida MD^48^, M. Macera MD^10^, E. Manzillo MD^46^, A. March MD^49^, D. Mascheroni MD^57^, A. Mastroianni MD^17^, I. Mauro MD^2^, M. Mazzitelli MD^23^, E. Mazzuca MD^11^, L. Mennella MD^16^, C. Micheletto MD^12^, A. Mingoli MD^29^, P. Minuz MD^21^, M. Moioli MD^14^, L. Monti MD^57^, R. Morgagni, MD PhD^9^, L. Mucci MD^58^, M. Muselli MD^47^, S. Negri MD^6^, C.G.A. Nobile MD^59^, S. Oldani MD^60^, C. Olivieri MD^27^, A. Papi MD^35^, G. Parati MD^61^, L. Parodi MD^31^, R. Parrella MD PhD^46^, E. Pastorelli MD^27^, V. Patruno MD^49^, F. Pellegrino MD^7^, P. Pelosi MD FERS^14^, M.F. Pengo, MD PhD^61^, D. Pepe MD^23^, A. Perotti MD^5^, R. Petrino MD^27^, M. Petrucci MD^30^, R.M. Piane MD^16^, G. Pignataro MD PhD^30^, M. Pino MD^23^, M. Pirisi MD^18^, V. Poletti MD^60^, F. Porru MD^62^, F. Pugliese MD^29^, R. Punzi MD^46^, D.A. Ramaroli MD^12^, C. Robba MD PhD^14^, R. Rostagno MD^27^, G. Ruocco MD^63^, U. Sabatini MD^28^, P.P. Sainaghi MD PhD^18^, F. Salton MD^34^, C. Salzano MD^64^, A. Sanduzzi MD^39^, S. Sanduzzi Zamparelli MD^10^, V. Sangiovanni MD^46^, D. Santopuoli MD^45^, P. Sapienza MD^29^, L. Sarmati MD^9^, E. Schiaroli MD^65^, F. Scienza MD^27^, M. Senni MD^4^, L. Serchisu PhD^51^, S. Sgherzi MD^57^, D. Soddu MD^18^, D. Soranna MD^61^, C. Sorino MD PhD^6^, S. Spadaro MD^35^, E. Stirpe MD^49^, C. Tana MD^58^, S. Tardivo MD^12^, S. Tartaglia MD^27^, E. Teopompi MD^20^, R. Terribile MD^27^, M. Tomchaney^1^, E. Torelli MD^30^, C. Torlasco MD^61^, C. Torti MD^23^, E. Tupputi MD^56^, C. Ugolinelli MD^5^, A. Vatrella MD^67^, A.G. Versace MD^8^, M. Villani MD^67^, L. Vincenzo MD^40^, C.A. Volta MD^35^, N. Voraphani MD^1^, J.C. Woods PhD^68^, E. Zekaj MD^69^, R. Zoppellari MD^35^, F.D. Martinez MD^1^

1. Asthma and Airway Disease Research Center, University of Arizona
2. Ospedale Scarlato, Scafati
3. ASST Bergamo Est, Seriate
4. ASST Papa Giovanni XXIII, Bergamo
5. Ospedale Sacco, Milano
6. ASST Lariana, Ospedale Sant'Anna di Como
7. Clinica San Carlo- Paderno Dugnano, Milano
8. Policlinico di Messina, Messina
9. Policlinico Universitario Tor Vergata, Roma
10. Università Vanvitelli, Napoli
11. A.O.O.R. Villa Sofia Cervello, Palermo
12. Università di Verona, Verona
13. Azienda Ospedaliera Universitaria di Padova, Padova
14. Policlinico San Martino and Universita’ di Genova, Genova
15. IRCCS Fondazione Don Carlo Gnocchi, Milano
16. Azienda USL Toscana Nord Ovest, Ospedale di Lucca
17. Azienda Ospedaliera di Cosenza, Cosenza
18. AOU Maggiore della Carità and Università del Piemonte Orientale UPO, Novara, Italy
19. Ospedale di Cremona, Cremona
20. AUSL-IRCCS RE Ospedale di Guastalla
21. Policlinico GB Rossi, Verona
22. Spedali Civili di Brescia, Brescia
23. Università degli studi Magna Graecia, Catanzaro
24. Ospedale San Giovanni di Dio, Crotone
25. AUSL-IRCCS, Reggio Emilia
26. Polytechnic University of Marche Region, Azienda Ospedali Riuniti, Ancona
27. Ospedale S Andrea, Vercelli
28. IRCCS Policlinico San Matteo Foundation, Pavia
29. Universita La Sapienza, Roma
30. Fondazione Universitaria Policlinico Gemelli - IRCCS. Università Cattolica del Sacro Cuore di Roma, Roma
31. Ospedale San Paolo, Savona
32. Ospedale San Giuseppe MultiMedica IRCCS- Milano
33. Ospedale di Eboli, Eboli
34. Ospedale di Trieste, Trieste
35. Universita' di Ferrara, Ferrara
36. Azienda Ospedaliera Pugliese Ciaccio, Catanzaro
37. Azienda Ospedaliera Universitaria Policlinico-Vittorio Emanuele, Catania
38. Ospedale Giulio Jazzolino, Vibo Valentia
39. Ospedale Monaldi and Federico II University, Napoli
40. ASST del Garda, Ospedale di Desenzano del Garda
41. Ospedale Rummo, Benevento
42. ASST Papa Giovanni XXIII, Bergamo, and University of Milan
43. Ospedale Dono Svizzero, Formia
44. Ospedale di Caserta, Caserta
45. Ospedale Cardarelli, Campobasso
46. Ospedale Cotugno, Napoli
47. Universita' de L'Aquila
48. Ospedale di Sassari, Sassari
49. Ospedale Universitario di Bolzano
50. ASL Salerno, Scafati
51. Ospedale di Bolzano, Bolzano
52. Ospedale di Perugia, Perugia
53. Azienda Sanitaria Locale and San Giuseppe Moscati Hospital, Avellino
54. Ospedale Santa Maria Goretti, Latina
55. ASST Mantova, Mantova
56. ASL BT. Pneumologia Andria
57. IC Villa Aprica, Gruppo San Donato, Como
58. Ospedale di Pesaro, Pesaro
59. Università della Calabria, Cosenza
60. Ospedale Morgagni, Forli’
61. IRCCS Istituto Auxologico Italiano, Ospedale San Luca, Milano
62. Erasmus MC, Rotterdam
63. Ospedale Regina Montis Regalis, Mondovì, Cuneo
64. Hospital "Buon Consiglio-Fatebenefratelli," Napoli
65. Ospedale di Perugia, Perugia
66. Universita’ di Salerno
67. Ospedale di Crema, Crema
68. Cincinnati Children’s Hospital and University of Cincinnati
69. IRCCS Istituto Ortopedico Galeazzi Milano
